# Addressing health inequalities: a case study on an oral health promotion program in New Caledonia

**DOI:** 10.3389/fpubh.2025.1636173

**Published:** 2025-10-02

**Authors:** Amal Skandrani, Helene Pichot, Estelle Pegon-Machat, Bruno Pereira, Stephanie Tubert-Jeannin

**Affiliations:** ^1^Centre de Recherche en Odontologie Clinique (CROC), UFR d'Odontologie, Université de Clermont Auvergne, Clermont-Ferrand, France; ^2^Health and Social Agency of New Caledonia (ASS-NC), Nouméa, France; ^3^Government of New Caledonia, Nouméa, France; ^4^Direction de la Recherche et de l'Innovation, Centre Hospitalier Universitaire de Clermont-Ferrand, Clermont-Ferrand, France

**Keywords:** health inequalities, health equity, social determinants, health promotion, oral health, indigenous peoples, health education

## Background

Disparities in oral health are deeply rooted in socio-environmental determinants that shape health behaviors and outcomes throughout life. These disparities appear from an early age and disproportionately affect indigenous peoples and socio-economically disadvantaged groups (1, 2). As a result, oral health serves both as a reflection of broader social inequalities and as a potential lever for social justice. The vision of health promotion set out in the Ottawa Charter recognizes the importance of addressing social determinants to achieve health equity, a principle aimed at ensuring equal opportunities for optimal health and well-being (3, 4). However, transforming these determinants requires more than targeted interventions; it requires fundamental change in the political, social, and economic power structures that perpetuate inequalities (5, 6).

As a French overseas territory located in the South Pacific, New Caledonia (NC) offers a unique perspective on the implementation of equity principles. NC currently enjoys an innovative status with broad autonomy and has authority over health matters. However, NC faces ongoing challenges related to socio-economic disparities and geographical isolation with a complex political landscape linked to the legacy of its post-colonial context. The territory's multi-ethnic population and geographical disparities lead to significant health inequalities. NC is divided into three provinces (North, South, Loyalty Islands), each characterized by distinct socio-economic conditions and varying access to healthcare. According to the 2019 census, 41% of the population identifies as Oceanian, 24% as European and 11% as Polynesian (7), with Oceanian people residing mainly in the North and Loyalty Islands provinces.

This article presents a case study that draws on field experience and epidemiological data to examine the outcomes of a territory-wide oral health promotion program (OHP) implemented in NC in 2014. Indeed, in the pursuit of health equity, research plays a crucial role in providing evidence on health inequalities, guiding policy decisions, and supporting the implementation and long-term evaluation of HP interventions (8). This case study therefore explores the program's results, the persistent challenges observed in the fight against health inequalities, particularly among indigenous and isolated populations, and examines future directions for equitable and effective HP policies in complex multicultural contexts (9). Key criteria and best practices for successful HP interventions are guiding this discussion (10).

## Program description

In 2012, an epidemiological study revealed high levels of oral diseases and obesity among children, highlighting shared risk factors and significant inequalities between provinces and ethnic groups (11, 12). In response, the New Caledonia Health and Social Agency, whose core mission of HP is publicly funded, launched the ‘Mes dents, Ma santé' (‘My Teeth, My Health') program as part of its efforts to improve general health and reduce health inequalities. This initiative aimed to promote oral health across the territory, particularly targeting disadvantaged areas. The program enhanced preventive dental care and encouraged collaboration with school health and educational teams within the national health plan (13). The program drew inspiration from international HP models, encompassing a range of interventions structured around three objectives: promoting healthy lifestyle habits, enhancing health education in schools, and strengthening integrated oral healthcare services (5, 14). Key components include HP campaigns, school-based health education, and preventive measures such as daily tooth brushing in schools and dental sealants for all children at age six (15). Adapted to the specificities of a multiethnic territory composed of three provinces with distinct governance structures, the program sought to ensure cultural relevance and institutional coordination.

## Assessing program outcomes with epidemiological data

Five years after the program was implemented, a cross-sectional follow-up study was conducted in 2019 with a representative sample of 1,474 schoolchildren aged 6, 9 and 12. Oral health status was assessed through clinical examinations, while questionnaires completed by the children and/or their parents provided additional information. This study provided valuable information on trends in oral health, provincial variations, associated risk factors and underlying social determinants. It also assessed the impact of oral diseases on children's quality of life.

Firstly, studies have shown a significant improvement in children's oral health between 2012 and 2019. Among 12-year-olds, the prevalence of untreated dental caries in permanent teeth decreased from 47% in 2012 to 30% in 2019 (Table 1). Geographical disparities persisted, with less progress in disadvantaged areas such as the Loyalty Islands than in the more prosperous south (the economic center and home to nearly 75% of the population), where the initial situation in 2012 was already more favorable. In the north, a disadvantaged region with high poverty rates, the implementation of the program was significantly strengthened by the participation of community health workers and educators, resulting in major improvements. The prevalence and severity of dental caries declined significantly, while progresses were limited in the Loyalty Islands, a province that is equally, if not more, disadvantaged. The prevalence of untreated caries declined only slightly, from 58% to 48.5%, and caries experience remained stable. The varied trends in dental status between provinces reflect the influence of social and environmental determinants, but also perhaps the different levels of the program' implementation (16).

**Table 1:** Changes in caries prevalences and indexes between 2012 and 2019 for 12 years old children, per province.

**Sample**	**South**		**North**		**Loyauty Islands**		**New Caledonia**	
	***N*** **(%)**		***N*** **(%)**		***N*** **(%)**		***N***	
2012	842 (70.1%)		249 (20.7%)		110 (9.2%)		1,201	
2019	459 (66.23%)		137 (19.77%)		97 (14%)		693	
**Prevalence**	***N*** **(%)**	**OR [IC]**	***N*** **(%)**	**OR [IC]**	***N*** **(%)**	**OR [IC]**	***N*** **(%)**	**OR [IC]**
**Prevalence for untreated dental caries (D**_3_**T**≠**0)**								
2012	371 (44.1%)		128 (51.6%)		64 (57.7%)		563 (46.9%)	
2019	122 (26.6%)	0.39 [0.19–0.79]^*^	42 (30.6%)	0.53 [0.28–1]^*^	47 (48.5%)	0.73 [0.38–1.38]	211 (30.5%)	0.46 [0.3–0.71]^*^
**Prevalence for teeth affected by dental caries (DM**_3_**FT** ≠ **0)**								
2012	479 (56.9%)		152 (61.3%)		67 (60.4%)		698 (58.1%)	
2019	178 (38.8%)	0.42 [0.22–0.79]^*^	56 (40.9%)	0.43 [0.24–0.76]^*^	55 (56.7%)	0.94 [0.46–1.92]	289 (41.7%)	0.47 [0.32–0.68]^*^
**Caries indexes**	**Mean** ±**SD**		**Mean** ±**SD**		**Mean** ±**SD**		**Mean** ±**SD**	
**D**_3_ **T index**								
2012	1.39 ± 40		1.82 ± 2.58		1.50 ± 1.80		1.49 ± 2.39	
2019	0.68 ± 1.52^*^		0.68 ± 1.35^*^		1.56 ± 2.01		0.79 ± 1.59^*^	
**D**_3_ **MFT**								
2012	2.06 ± 2.86		2.34 ± 2.93		1.65 ± 2.05		2.09 ± 2.82	
2019	1.15 ± 2.02^*^		1.07 ± 1.82^*^		2.04 ± 2.52		1.26 ± 2.08^*^	
**SIC**								
2012	5.45 ± 2.93		5.52 ± 2.62		4.28 ± 1.90		5.37 ± 2.80	
2019	3.91 ± 2.20^*^		3.6 ± 2.00^*^		4.49 ± 2.07^$^		3.97 ± 2.15^*^	

OR odd ratio with IC = 95% confidence interval.

D_3_MFT, number of permanent teeth affected by dental caries (decayed, missing or filled).

D_3_, decayed teeth diagnosed at the dentinal/cavity stage.

SIC, average DMFT for the most affected third of the population.

^*^p < 0.05 multilevel mixed effects generalized linear models (random effects considering investigator and school effects).

^$^NaN, not a number. Significant Caries Index (SIC).

Beyond territorial differences, the 2019 epidemiological study confirmed the persistence of ethnic disparities. For the 9-year-old group, a Zero-Inflated Negative Binomial regression was conducted, with the number of untreated carious lesions as the primary outcome variable. Explanatory factors were categorized into three main categories: socio-demographic characteristics, access to oral health care, and oral health behaviors. Variables were included in the multivariate models based on their statistical significance in the bivariate analyses and their clinical relevance. Results revealed that significant factors operated independently across these categories: (1) social determinants, with Oceanian children being at higher risk compared to other ethnic groups; (2) access to oral healthcare, as children without sealed molars or reporting accessibility problems showed poorer oral health; and (3) lifestyle habits, with high consumption of free sugars and low toothbrushing frequency having a significant impact on dental status (17).

The 2019 study also revealed the broader impact of oral diseases on children's Oral Health related Quality of Life. Indeed, the French version of the COHIP questionnaire had been filled by the 12-year-olds children. It was found that 61% of the children reported functional difficulties (e.g., eating, speaking, brushing), 64% experienced emotional distress (e.g., anxiety, sadness), and 25% missed school at least once due to dental problems. Notably, these impacts were more frequent among Oceanian children and those from the Loyalty Islands. The results suggest that oral health issues extend well beyond physical discomfort, undermining children's well-being, self-esteem, and educational continuity (18).

## Lessons learned

### Program enablers and achievements

The program led to tangible improvements in children's oral health, particularly in areas where implementation was effective. The program emphasizes evidence-based preventive measures, daily tooth brushing in schools, fissure sealants applications and oral health education. Health education was delivered through an approach that addressed both oral health behaviors and health literacy, with the aim of empowering families and the broader community. Evaluation results indicated strong community engagement, with in 2018, 65% of primary schools that had implemented supervised tooth brushing and 91% of six-year-old children who benefited from fissure sealants' applications (19, 20).

The systematic collection of epidemiological data has also been instrumental in informing strategic planning, tailoring interventions to local contexts, monitoring both implementation processes and health outcomes. Data collection was intended to identify needs, implement adapted actions and to evaluate health outcomes, aligning with international recommendations for the search of equity in health promotion (3). Some qualitative assessments were also conducted to gain deeper insights into the program's level of acceptability and the extent of community engagement.

### Structural barriers to reducing health inequalities

Despite encouraging results attributable to the program, its uneven impact highlights the persistent challenges of implementing effective and equitable HP interventions, given that health inequalities are mainly socially constructed within an environmental context. Several structural factors have limited the reach and effectiveness of the program in vulnerable areas.

Geographic isolation and logistical constraints were particularly pronounced in the Loyalty Islands, where supply chain issues, limited infrastructure and absence of locally based facilitators hindered the deployment of school-based interventions. In contrast, the North Province better supported preventive interventions, mobilizing health educators and community health auxiliaries to ensure consistent implementation and follow-up. Institutional fragmentation also played a role. Health governance is decentralized across three provinces with differing capacities, resources, and levels of political engagement. While this allows for contextual adaptation, it also creates disparities in the prioritization, funding, and operational support of public health initiatives.

Unequal distribution of the oral health workforce further exacerbated these challenges. Approximately 82% of dentists practice in the South, mostly in private practice, where infrastructures are more developed. In contrast, only 14% and 4% of dentists work in the North and Loyalty Islands, respectively, with most of them serving in public health centers. These disparities limit access to oral health services in remote and disadvantaged areas.

These structural barriers, whether social, cultural, logistical or institutional, are deeply rooted in the local context and it has not been possible to change them fundamentally. However, they play a key role in the ability of OHP programs to reach their full potential for acting on health inequalities.

## Recommendations to enhance health and equity

OHP programs typically rely on combined interventions: creating supportive environments through health policy, strengthening health literacy, empowering communities, and ensuring access to health services (4, 21).

Indeed, political support is essential for creating more favorable living environments and mitigating social and commercial determinants of health. Including OHP in the “Do kamo” health plan has been a key milestone in the development of the program. However, it is regrettable that many upstream interventions have been delayed or even abandoned. For instance, a sugary drinks tax -an evidence-based effective measure -was only implemented in 2024 at low rates (22, 23). Other measures included harmonizing the sugar content of drinks by aligning regulations and nutrition standards with those of France. These have yet to be enacted, undermining the effectiveness of broader preventive efforts aimed at reducing the incidence of dental caries. This demonstrates the importance of commercial determinants of health; preventive and educative interventions struggle to counterbalance the fact that most children frequently consume sugary foods and drinks outside of meals, particularly local soft drinks with very high sugar content.

Achieving health equity also necessitates a participatory approach that prioritizes cultural relevance and community ownership (24). Indeed, in areas like New Caledonia, which have a colonial history and significant cultural diversity, fostering genuine co-construction with Indigenous communities has been shown to be key to ensuring equity (25–27). In the present case, while Caledonian imagery was integrated into educational and communication materials and a qualitative study explored barriers to dental care—revealing widespread self-medication, high dental anxiety, and negative perceptions of dentists, alongside positive attitudes toward preventive care for children -, public participation in decision-making processes has remained limited (28). To cultivate more meaningful engagement, traditional knowledge and community-led approaches should be better embedded throughout the program cycle, from design to evaluation. This includes co-developing oral health messages with customary leaders, incorporating local beliefs and practices into educational content, and enriching training programs for health personnel with local health practices and community-based perspectives. Participatory evaluation reflecting local worldviews and social representations of health could also enhance legitimacy and responsiveness.

To address implementation gaps within a territory or a population, it is not enough to simply intensify existing efforts (29). Notably, our findings highlighted the need for more targeted efforts in the Island Province and among Oceanian populations. Ensuring access to health services for all, requires the development of strategies tailored to underserved areas, as well as the establishment of governance mechanisms that link centralized and local management, rooted in the realities of each context. In this regard, the use of a proportionate universalism approach, in which interventions are tailored to the level of need, seems particularly relevant. Previous studies, such as in Australia, have highlighted the value of targeting high-risk populations and of co-developing culturally appropriate preventive interventions to prevent oral diseases in an equitable manner (30).

Decolonizing OHP requires going beyond cultural adaptations toward genuinely community-led design where local communities are empowered as co-constructors of health promoting interventions and related reporting of health research, rather than being passive recipients (31). Assessing whether an HP program challenges or perpetuates power structures, such as unequal access to oral health services is also key to help ensure health equity (32). This kind of transformation is essential to evolve from a promising initiative into a sustainable, equity-driven public health success.

## The challenge of long-term sustainability

The sustainability of OHP interventions is heavily influenced by the political context, with upheavals often threatening program continuity (33). For instance, the COVID-19 pandemic disrupted health systems by shifting priorities to immediate crisis management. Similarly, the 2024 political crisis in New Caledonia marked by riots and violence heightened the risk of program termination, especially as healthcare facilities were targeted, leading to a significant reduction in oral health care availability (34). Thus, restoring political stability is essential for the future of HP programs.

## Conclusion

This case-study highlights the multiple challenges involved in implementing OHP programs. Although the program has achieved notable progress over the past 5 years, persistent health disparities reveal its limitations. Advancing toward greater equity would require not only addressing structural barriers but also better embracing the principles of knowledge decolonization and Indigenous-informed strategies. Building on this foundation, strong political commitment will be essential to ensure the future of a renewed program, supported by regular epidemiological studies and qualitative research involving indigenous peoples, and economic evaluations to support equitable, effective and economically sustainable interventions (35).
